# Immunoregulatory Roles of Extracellular Vesicles and Associated Therapeutic Applications in Lung Cancer

**DOI:** 10.3389/fimmu.2020.02024

**Published:** 2020-08-28

**Authors:** Zhengrong Yin, Jinshuo Fan, Juanjuan Xu, Feng Wu, Yang Li, Mei Zhou, Tingting Liao, Limin Duan, Sufei Wang, Wei Geng, Yang Jin

**Affiliations:** NHC Key Laboratory of Pulmonary Diseases, Department of Respiratory and Critical Care Medicine, Union Hospital, Tongji Medical College, Huazhong University of Science and Technology, Wuhan, China

**Keywords:** extracellular vesicles, lung cancer, immunosuppression, immunostimulation, therapeutic application

## Abstract

Lung cancer represents a fatal condition that has the highest morbidity and mortality among malignancies. The currently available treatments fall short of improving the survival and quality of life of late-stage lung cancer patients. Extracellular vesicles (EVs) secreted by tumors or immune cells transport proteins, lipids, and nucleic acids to other cells, thereby mediating immune regulation in the tumor microenvironment. The cargo carried by EVs vary by cellular state or extracellular milieu. So far, multiple studies have suggested that EVs from lung tumor cells (TEVs) or immune cells promote tumor progression mainly through suppressing antitumor immunity. However, modified or engineered EVs can be used as vaccines to elicit antitumor immunity. In addition, blocking the function of immunosuppressive EVs and using EVs carrying immunogenic medicine or EVs from certain immune cells also shows great potential in lung cancer treatment. To provide information for future studies on the role of EVs in lung cancer immunity, this review focus on the immunoregulatory role of EVs and associated treatment applications in lung cancer.

## Introduction

Lung cancer remains the most frequently diagnosed cancer, accounting for 11.6% of total cases, and a leading cause of cancer-related mortality, responsible for 18.4% of total cancer deaths around the globe (1) with its 5-year survival being 19% (2). Most lung cancer patients, especially late-stage patients, have unfavorable prognoses due to delayed diagnosis and unresponsiveness to conventional therapies (surgery, chemotherapy, and radiotherapy) (3). Recently, mounting attention has been paid to immunotherapy for its good curative effects. For instance, chimeric antigen receptor (CAR) T-cells can successfully eradicate hematologic malignancies, but their effect on solid tumors, including lung cancer, has not been satisfactory (4). Treatments involving immune checkpoint inhibitors (such as programmed death-1 (PD-1) inhibitor, programmed death-ligand 1 (PD-L1) inhibitor, and cytotoxic lymphocyte antigen 4 (CTLA4) inhibitor) are remarkably effective in many malignancies, including lung cancer. Nonetheless, only about 20% of lung cancer patients respond to these therapies, and the efficacy largely depends on the adequate expression of PD-1 or PD-L1 on the immune or cancer cells (3, 5). These advances indicate that immunotherapy has great potential in the treatment of lung cancer, and further breakthroughs are needed. We recently found that autologous lung tumor–derived microparticles (ATMPs) loaded with methotrexate (ATMP-MTX) had a good safety profile and were effective for the treatment of advanced lung cancer with malignant pleural effusion (6). EV-associated therapy for lung cancer has become a research hot spot and may result in a major breakthrough in the treatment of lung cancer in the future (7, 8).

Extracellular vesicles, a collective term for various membrane structures released by virtually all cells principally involve exosomes, microparticles, and apoptotic bodies (9). EVs contain a variety of cargo from donor cells, including proteins, lipids, and genetic substances (10, 11). The cargo can be transferred by EVs to recipient cells, resulting in phenotypic changes in the latter and vice versa (12). Cargo possessing distinct properties are selectively enriched within different subtypes of EVs from various donor cells under specific ambient conditions (13, 14). As a medium that mediates cell-to-cell communication, EVs are implicated in a wide array of biological activities in malignancies, including lung cancer (12, 15). These activities include metastasis (16), angiogenesis (17), and regulation of host immune function (15, 18). Currently, immune regulation of EVs is a hot spot of related research. Multiple studies have reported that EVs from both cancer and immune cells are involved in the immune regulation of lung cancer (11, 18). More specifically, natural EVs mainly play an immunosuppressive role in various cancers, including lung cancer (19–23) although, on the other hand, modified EVs may serve as activators of antitumor immunity (6, 24–26).

Accordingly, EV-associated therapies have been tried in the treatment of lung tumors (6, 11, 18, 19, 21, 24–35). EVs involved in these studies range from modified or engineered TEVs and DEVs to EVs from embryonic stem cells (ESEVs) and T-lymphocytes (LEVs). Discrepancies in efficacy among these trains of research can mainly be ascribed to differences in study design, such as the different subtypes of EVs used. Therefore, there is urgent need to comprehensively review and characterize the immunological features of EVs and to make full use of previous research findings concerning EVs to develop effective treatment methods for lung cancer. In this paper, we principally review the roles of EVs as mediators of intercellular communication between tumor and immune cells in the modulation of antitumor immunity and relevant therapeutic applications in lung cancer.

## Biogenesis of EVs

The existence of vesicles in the extracellular milieu in mammalian tissues or fluids was first described in the late 1960s, and since then, mounting attention has been paid to their roles (36, 37), and remarkable results have been accomplished regarding EVs over the past decades. “Extracellular vesicles,” as a generic term, was recommended by the International Society for Extracellular Vesicles (ISEV) to refer to the “particle released from the cells that are delimited by a lipid bilayer and cannot replicate.” It is so defined mainly because, up to now, no consensus has been reached among researchers concerning the specific markers for various subtypes, mainly including exosomes, microparticles/microvesicles/ectosomes, oncosomes, and apoptotic bodies (9, 38). In terms of the assembling and releasing process, it has now been generally accepted that “ectosomes” (microparticles/microvesicles) are formed by outward budding of the plasma membrane, exosomes derive from fusion of multivesicular bodies (MVBs) with the plasma membrane (39, 40), and apoptotic bodies are generated by cells undergoing apoptosis (40, 41). In different biogenetic processes, certain substances are selected and enriched within specific subtypes of EVs, and constitution of cargo in one type of EV might change as the microenvironment of donor cells changes (13, 14, 40). Accordingly, particular cargo dictates the properties of various subtypes of EVs, which indicates that different EV subtypes play variant roles in tumor immunomodulation (13, 14).

## Immune Cargo of EVs

The proportion of EVs secreted by a cell varies depending on the donor cell type and its state (42). The production of a certain subtype of EV also changes with the transformation of donor cells (43, 44). The bioactive molecules in EVs are derived from cell membranes and endosomes (12, 39), and the uniqueness of these molecular characteristics is related to the donor cell (45). EVs produced by lung tumor cells or immune cells contain plenty of bioactive substances, such as proteins, lipids, and genetic DNA/mRNA/non-coding RNA, which are transported between cells and can deliver information about immune processes (11). In most cases, EVs from lung tumor cells contain certain cargo that induces immune escape (19, 21, 46, 47). Nonetheless, after modification, immunogenic components (e.g., tumor-associated antigens) on EVs derived from tumor cells gradually function and can activate antitumor immunity (24–26, 48, 49). Similarly, EVs derived from immune cells contain many functional molecules and mediate communication among immune cells (43, 50–54). Additionally, EVs from embryonic stem cells also possess some antigens similar to those from cancer cells (31). All immunosuppressive and immunostimulatory cargo in EVs discussed in this review are listed in Tables 1, 2, respectively.

**TABLE 1:** Immunosuppressive cargo of EVs in lung cancer.

Cargo	EV types	Modifications or treatments	Mechanisms	Functions in immunity	References
NA	TEXs	\	IL-6/STAT3^∧^	Inhibiting the maturation of DCs	(19)
NA	TEXs	\	NA	Inducing Treg cells	(19)
NA	TEXs	\	NA	Decreasing c-c/c-x-c chemokine receptor on DCs	(19)
NA	TEXs	\	NA	Upregulating immunosuppressive molecules on DCs	(19)
NA	TEXs	\	NA	Downregulating immunostimulatory molecules on DCs	(19)
PD-L1	TEXs	\	PD-L1/PD-1	Inducing anergy or apoptosis of T cells	(20)
HSP72/HSP70	TEXs	\	TLR2/MyD88/IL-6/STAT3	Activating immunosuppressive function of MDSCs	(21)
Non-coding RNAs	TMPs	UV-irradiation	TLR3/IL-1β	Increasing secretion of IL-1β from M2 type macrophages and promoting tumor progression	(47)
DNAs^∧^	TMPs	UV-irradiation	cGAS/STING/TBK1/STAT6	Inducing M2 polarization of macrophages	(78)
U1snRNA	TMPs	\	TLR3	Inducing tumor-promoting inflammation	(22)
FasL	LMPs	Activation	APO2L/TRAIL; Fas/FasL	Inducing death of T cell; Inducing apoptosis of DCs	(81, 82) (23)

*NA, not available; ^∧^, uncertain; \, none; EVs, extracellular vesicles; TEXs, Lung cancer-derived exosomes; IL-6, interleukin-6; STAT3, signal transducer and activator of transcription 3; DCs, dendritic cells; Treg cells, regulatory T-cells; PD-1, programmed death-1; PD-L1, programmed death-ligand 1; HSP72/HSP70, Heat Shock Proteins 70/72; TLR2, toll-like receptor 2; MyD88, myeloid differentiation factor 88; MDSCs, Myeloid-derived suppressor cells; TMPs, lung tumor-derived microparticles; UV, ultraviolet; TLR3, toll-like receptors 3; IL-1β, interleukin-1β; cGAS, Cyclic GMP-AMP synthase; STING, stimulator of interferon gene; FasL, Fas ligand; LMPs, T lymphocytes-derived microparticles; APO2L, Apoptosis ligand 2; TRAIL, tumor necrosis factor-related apoptosis-inducing ligand.*

**TABLE 2:** Immunostimulatory cargo of EVs in lung cancer.

Cargo	EV types	Modifications or treatments	Mechanisms	Functions in immunity	References
CCL2-5, CCL20	TEXs	Heat treatment	CCL/CCR	Recruiting and stimulating CD11c^+^DC and CD4^+^/CD8^+^ T-cells	(25)
CD54, CD86	TEXs	Heat treatment	NA	Adhering to DCs	(25)
MHCI/II	TEXs	Heat treatment	NA	Antigen presentation	(25)
NA	TEXs	Rab27a overexpression	NA	Inducing maturation of DCs	(26)
CD40L	TEXs	CD40L-engineering	CD40/CD40L	Inducing maturation of DCs	(49)
Her2/neu, CEA, WT1, MAGE2, and survivin peptides	Lung tumor-derived apoptotic bodies	MAGE-engineering and UV-irradiation	NA	Presenting antigens to DCs and stimulating DCs maturation	(28)
MAGE peptides	DEXs	DEX incubated with MAGE peptides	pMHCII costimulatory molecules/TLR	Antigens cross-presentation among DCs, NK cell activation	(29)
Certain carcinoembryonic antigens	ESEXs	GM-CSF engineering	NA	Inducing antigen-specific antitumor immune response	(31)
MTX	LMPs	MTX-incorporation and UV-irradiation	NA	Inducing immunogenic death of lung cancer cells	(6)

*NA, not available; CCL, chemokine (C-C motif) ligand; CCR, C-C motif chemokine receptor; MHCI/II, major histocompatibility complex I/II; Her2/neu, human epidermal growth factor receptor 2; CEA, carcinoembryonic antigen; WT1, Wilms’ tumor gene 1; MAGE, melanoma antigen gene; pMHCII, peptide-MHCII; BAG6, BCL2-associated athanogene 6; NKp30, natural killer (NK) cell receptor; ESEXs, exosomes from embryonic stem cells; GM-CSF, granulocyte-macrophage colony-stimulating factor; MTX, methotrexate.*

## Lung Tumor Immune-Microenvironment (TIME)

The TIME of lung cancer has to be mentioned before we discuss the immunoregulatory roles of EVs because EVs are just one of the mediators of immune regulation in the TIME.

Tumors are considered to be caused by genetic mutations that may generate neoantigens and trigger immune surveillance to clear or suppress non-self tumor cells. However, this self-protection mechanism frequently fails as cancer develops (55). The cancer immunoediting hypothesis, designed to explain this phenomenon, assumes that there exist three phases during the process of tumorigenesis and tumor progression, namely “elimination,” “equilibrium,” and “escape” (56). This complicated process is actually a battle between cancer cells and the host immune system. In the “elimination” phase, the host immune system prevails, whereas during the “escape” phase, cancer cells defeat the host immune system. “Equilibrium” refers to a standoff between the two sides. The entry into the “escape” phase involves an interaction among various players, including tumor cells, tumor stroma, and the host immune system (56). Briefly, the negative results of this “struggle” include impaired immune recognition (such as loss of tumor antigens), increased resistance to the cytotoxic effects of immunity, or especially, establishment of an immunosuppressive state within the tumor microenvironment (56). The formation of the immunosuppressive microenvironment involves immunosuppressive cytokines (such as vascular endothelial growth factor (VEGF), transforming growth factor-β (TGF-β), galectin, or indoleamine 2,3-dioxygenase (IDO)] released by cancer cells; these cytokines block the maturation of immune cells and promote the recruitment of immunosuppressive cells (57).

Research looking into the maturation of dendritic cells (DCs) from lung cancer biopsies shows that DCs consist of three types, i.e., CD11c^high^ mDCs, CD11c^–^ pDCs and CD11c^int^ mDCs, in terms of their expression levels of CD11c, and most tumor-infiltrating DCs (TIDC) are “semi-mature” or even immature (58). Moreover, those TIDCs isolated freshly from non-small cell lung cancer (NSCLC) underwent only slight phenotypic maturation and showed poor antigen-presenting ability after toll-like receptor (TLR) activation *in vitro* (58). Increased proportions of CD4^+^CD25^+^ Treg cells secreting TGF-β were found in tumors and peripheral blood from patients with lung cancer, and tumor-infiltrating lymphocytes showed only marginal production of Th1 or Th2 cytokines (59). Macrophages in tumor tissue can be stimulated by tumor-derived cytokines and polarized into the M2 type. The latter could subvert adaptive immunity and promote tumor progression (60). Secretion of EVs with suppressors of cytokine signaling 3 (SOCS3) from alveolar macrophages were inhibited in patients with non-small cell lung cancer and in a lung cancer mouse model, which promoted the development of lung tumors (61). Myeloid-derived suppressor cells (MDSCs) are a group of phenotypically heterogeneous immature cells of bone marrow origin and have a remarkable ability to suppress T-cell activation (62). These cells were found to be more suppressive and apparently increased in peripheral blood, tumor tissues, spleen, and lymph nodes in tumor-bearing mice or humans compared to normal controls (62).

In summary, lung cancer cells will do all in their power to escape from host antitumor immunity for their survival. Understanding this essential concept can help us decipher the roles of EVs in lung cancer immunity.

## Roles of EVs in Immunoregulation in Lung Cancer

As an efficient medium conveying information between cells, EVs contain specific antigens or immune molecules from tumor or immune cells, and they play a vital role in cancer immunoediting (11). EVs produced by tumor cells can be internalized by immune cells, thereby altering the function of immune cells and vice versa.

**FIGURE 1:**
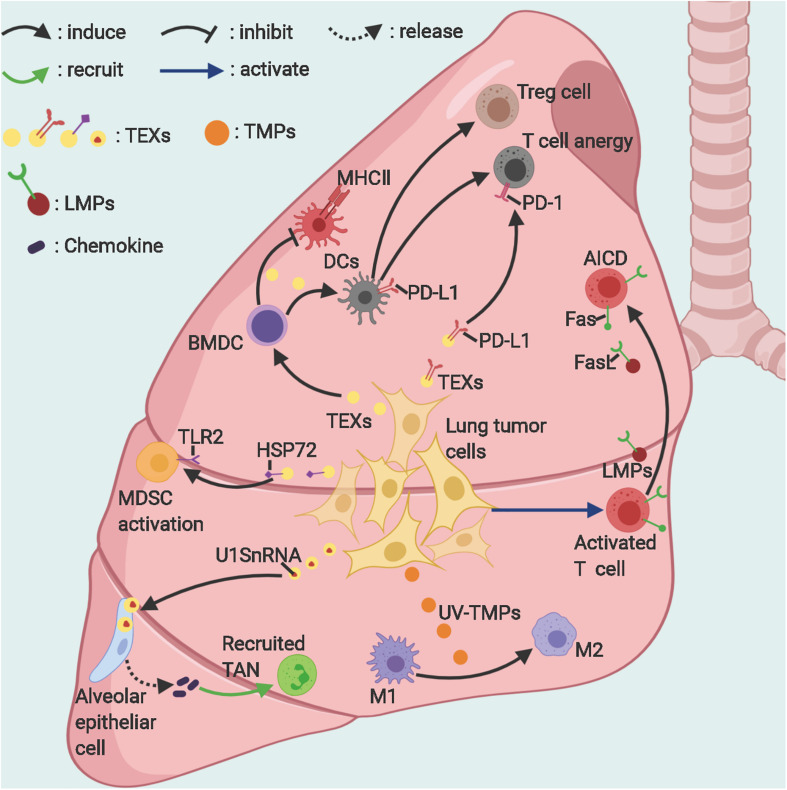
The suppressive roles of EVs in lung cancer immunity. Lung tumor–derived exosomes and microparticles can suppress antitumor immunity in various ways. Activated T-cells release microparticles, which induce their own death via Fas/FasL signaling. *TEXs, Lung tumor-derived exosomes; TMPs, tumor-derived microparticles; LMPs, T-lymphocyte-derived microparticles; TAN, tumor-associated neutrophil; AICD, activation-induced cell death; MDSCs, Myeloid-derived suppressor cells; M1/2, macrophages subtype 1/2*. Figure created with BioRender.com.

In almost all TIMEs, EVs act as an immunosuppressor (19–23) (Figure 1). More precisely, in the process of immunoediting, EVs may serve as an immune stimulator at the germination of cancer cells (which may not go through an immunoediting process) and then convert to an immunosuppressor during the progression of cancer. A classical research, though not studying lung cancer cells, demonstrates that exosomes from poorly metastatic melanoma cells can potentially inhibit cancer metastasis to the lung by stimulating an innate immune response and triggering cancer cell clearance at the pre-metastatic niche (63) while exosomes from advanced and highly metastatic melanoma help create pre-metastatic niches in remote microenvironments to favor metastasis (64).

It is worth noting that TEV-mediated tumor immunoregulation is closely related to TEV-mediated tumor metastasis; the latter is a complicated process dubbed the infiltration-metastasis cascade (65). On the one hand, TEVs activate epithelial-mesenchymal transition (EMT) in neoplastic, mesothelial, and vascular endothelial cells through various signaling pathways (such as TGF-β, Wnt5b, or caveolin-1 signaling pathways), thereby enhancing the migration ability of cancer cells and increasing the permeability of blood vessels in the peritumoral matrix (65–69). Particularly, in addition to metastasis promotion, TGF-β also plays an important role in immune regulation (70) as TGF-β could induce fibroblasts to release the immunomodulatory protein PD-L1 into extracellular vesicles, resulting in inhibition of T-cell proliferation. At the same time, PD-L1 knockdown could reduce the induction of TGFβ-dependent extracellular matrix protein production and, thus, suppress cell migration (71). On the other hand, TEVs can be systemically transported to distant locations, thereby fostering a pre-metastatic niche via activating a reactive, myofibroblast-rich stroma and promoting immune evasion (65, 68, 72). Therefore, the immunosuppressive action and metastasis-promoting effect of EVs complement each other to promote tumor progression.

**FIGURE 2:**
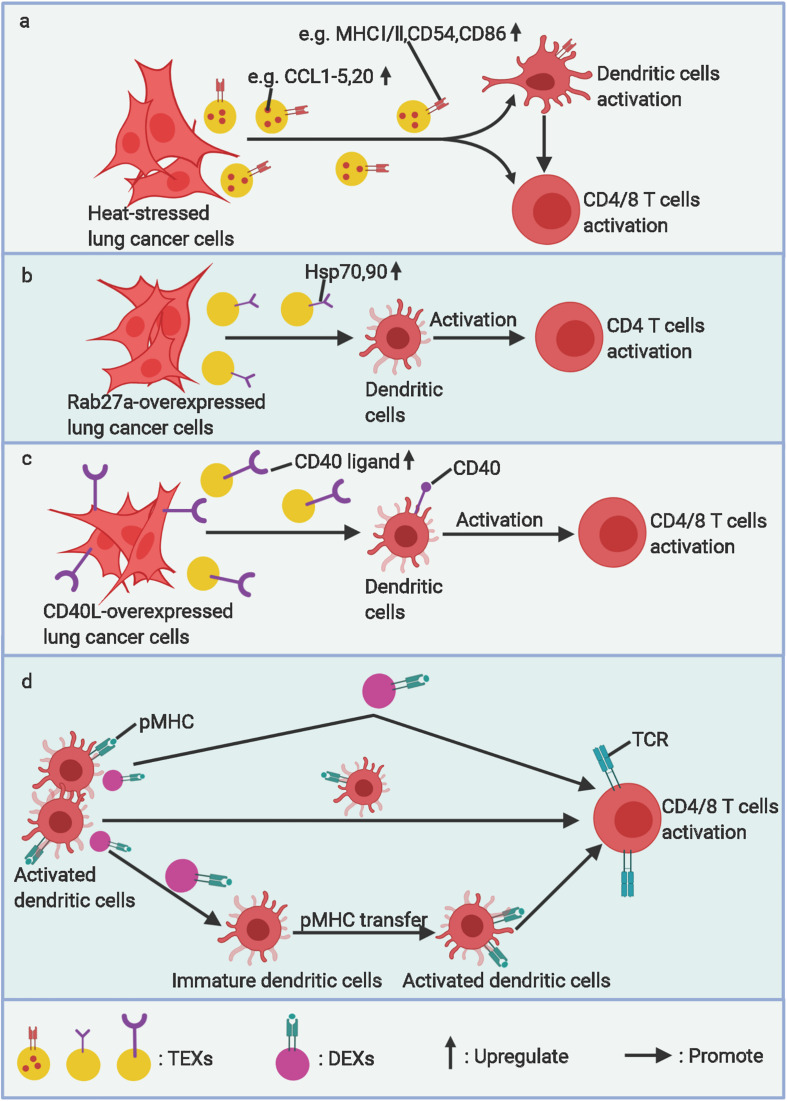
The stimulatory roles of EVs in lung cancer immunity. **(a)** Heat stressed, **(b)** Rab27a overexpressed, and **(c)** CD40L overexpressed lung cancer cell–derived exosomes (TEXs) can activate DC maturation and arouse specific antitumor immunity. **(d)** Activated dendritic cell–derived exosomes (DEXs) can present antigens to T-cells directly or by transferring pMHC to other immature dendritic cells and amplifing this antigen-presentation effect. *TCR, T-cell receptor*. Figure created with BioRender.com.

Nevertheless, other researchers found that, under certain stresses (such as exposure to radiation, heat, or ultraviolet light) or when engineered with a specific aptamer, reconstructive tumor and immune cells can release corresponding EVs with a stronger immune-stimulating property but reduced immunosuppressive ability (6, 24–26) (Figure 2). Anticancer immunotherapy using EVs is precisely premised on these findings.

In this section, we mainly discuss the immunosuppressive roles of natural TEVs and T-lymphocyte–derived MPs (LMPs) in lung cancer and the immunostimulatory evidence of processed or engineered TEVs and DEVs. Knowing these regulatory mechanisms is pivotal for developing optimal protocols to use EVs to effectively elicit antitumor immunity.

### Immunosuppressive Roles

#### Natural EVs From Lung Cancer Cells

##### Inhabiting function of dendritic cells

It has been demonstrated that exosomes derived from Lewis lung cancer (LLC) cells could block the differentiation and maturation of myeloid precursors into DCs and induce apoptosis of myeloid precursors in the presence of FLT-3L *in vitro* (19) as indicated by decreased CD11c^+^ DCs and downregulated maturation markers of CD80/CD86/MHCII on DCs (19). The underlying mechanism has not been specifically explored by any studies, but it can be speculated on the basis of findings of other similar studies concerning other cancers. For instance, research shows that the exosomes from murine or human breast cancer cells could block the differentiation of murine myeloid precursor cells into immature CD11c^+^ DCs by inducing expression of interleukin-6 (IL-6) and activating the signal transducer and activator of transcription 3 (STAT3) (46).

LLC exosomes can induce the expression of immunosuppressive molecules, including PD-L1, CD11b, and Arginase I, and downregulate the expression of immune activating/stimulatory molecules, such as CD80, CD86, and MHC-II on dendritic cells (19). These treated DCs also decrease the mRNA level of certain immunocompetent molecules, such as tumor necrosis factor-α (TNF-α), IL-6, and inducible nitric oxide synthase (iNOS), and the aforementioned changes in DCs eventually lead to T-cell anergy (19). A PD-L1-blocking antibody can partially eliminate the inhibitory effect of DCs treated by LLC exosomes rather than 4T1 exosomes, indicating that other molecules, rather than PD-L1 on DCs treated by the 4T1 exosome, mediate the immunosuppressive effects (19).

Lung cancer (LLC)-derived exosomes (TEXs) inhibit migration of DCs to lymph nodes by decreasing most C-C/C-X-C chemokine receptors, especially CCR6, CCR7, and CXCR3 on DCs (19). These TEXs inhibit the migration of DCs to draining lymph nodes and block the interaction between DCs and T-lymphocytes. However, little is known about what substances on TEXs mediate this effect and how they work.

##### Induction of apoptosis of T-cells

PD-L1 was found on exosomes as well as donor lung cancer cells, and these PD-L1-expressing exosomes can suppress cytokine secretion and induce anergy or apoptosis of PD-1-expressing activated T-cells (20). The mRNA level of PD-1 in circulating exosomes of patients with NSCLC was found to be significantly associated with the effect of anti-PD-1 therapy (73). Indeed, it was demonstrated that a high level of PD-L1-expressing TEXs in blood, as a variant of secreted PD-L1, could neutralize the administrated anti-PD-1 antibodies (74) before they reached tumor tissues, thereby resulting in poor response and outcomes (75).

##### Induction of immunosuppressive immune cells

FOXP3-expressing regulatory T (Treg) cells, as a significant component maintaining immune homeostasis, also suppress antitumor immunity. The infiltration of FOXP3^+^Treg cells into a tumor was found to be highly related to poor prognosis of cancer patients. TEX-treated DCs were shown to induce the differentiation of CD4^+^FOXP3^+^Treg cells while suppressing the differentiation of CD4^+^IFN-γ^+^ Th1 cells (19).

Various factors in a tumor could induce the expansion or activation of MDSCs through multiple pathways, including STAT3 or IL-4Rα–STAT6 pathways, resulting in the suppression of T-cell function (62). EVs have been increasingly shown to take part in the communication between tumor cells and MDSCs. Research in mice and humans shows that TEXs (including lung cancer exosomes) can activate immunosuppressive function of MDSCs (21). Specifically, heat shock protein 72 (Hsp72) expressed on TEXs interacts with the TLR2 on MDSCs, which triggers the TLR2/MyD88 signaling pathway, induces the autocrine production of IL-6, and causes Stat3 phosphorylation, which ultimately activates the suppressive function of MDSCs (21). Of note, TEXs induce the activation of Stat3 without promoting MDSC expansion while the tumor-derived soluble factors (TDSFs) trigger the expansion of MDSCs by activating the Erk signaling pathway (21). On the whole, these results are supplementary to the findings that regulation exists between tumor cell and MDSCs and are conducive to the further understanding of the development of MDSCs against a tumor background.

Additionally, TEXs were also found to induce tumor-promoting inflammation in sites far from the primary tumor and to mediate tumor metastasis (22). U1 snRNAs in exosomes derived from LLC or B16/F10 melanoma cells can be transferred to alveolar epithelial cells and could be sensed by TLR3. Activated TLR3 signals increase the production of chemokines, which promotes the recruitment of neutrophils to the lung. These infiltrating neutrophils would be polarized into tumor-promoting subtypes (tumor-associated neutrophils, TANs) (76, 77) and eventually enhance the formation of a pre-metastatic niche in the lung (22). The mechanism is akin to that by which the non-coding RNA in lung tumor–derived microparticles (TMPs) stimulates TAM to secrete IL-1β as described below (47).

#### MPs From Ultraviolet (UV)-Treated Lung Cancer Cells

MPs from UV-treated lung cancer cells could induce polarization of M2 macrophages to suppress antitumor immunity and promote tumor progression *in vitro* and *in vivo* (47, 78). Specifically, macrophages treated with MPs from UV-treated lung cancer cells upregulate the expression of M2-type surface markers CD163, CD206, VEGF, IL-10, and arginase 1 and downregulate the level of M1-type surface markers IL-12, iNOS, and TNF-α (47, 78). The mechanism of MPs stimulating M2 type macrophages and promoting tumor progression involves the activation of cGAS/STING/TBK1/STAT6 pathways in macrophages (78). Furthermore, whether DNAs in these TMPs activate the cGAS/STING pathway in M2 macrophages needs further investigation (78). Meanwhile, the tumor-promoting effects of M2 macrophages induced by lung cancer microparticles are associated with increased IL-1β secretion after macrophages sense the non-coding RNA in TMPs through the TLR3 signaling pathway (47). IL-1β has been proven to promote the angiogenesis (79) and stemness of tumor cells (80).

#### EVs From Activated T-Lymphocytes

Activated T-cells could release microvesicles carrying Fas ligand (FasL) and apoptosis ligand 2 (APO2L), which mediates the activation-induced cell death (AICD) of mature T-cells (81, 82). Additionally, exosomes from mature T-cells can be taken up or internalized by DCs via exosomal LFA-1, thereby downregulating the expression of peptide/MHC I on DCs and inducing the apoptosis of DCs via the Fas/FasL signaling pathway (23).

### Immunostimulatory Roles

#### Modified EVs From Lung Cancer Cells

Although natural TEVs mainly mediate immunosuppressive roles in lung cancer as aforementioned, TEVs do share similar antigens with donor tumor cells as indicated by their ability to induce a tumor-specific immune response (25). Mounting evidence suggests that EVs from stress-treated (25) or modified (49) lung cancer cells with stronger immunogenicity can activate DC maturation and specific T-cell immune response (Figures 2a–c). Some artificially immunogenicity-enhanced TEVs used for stimulating immunity are detailed in following paragraphs.

Heat-stressed 3LL lung tumor cell–derived exosomes (HS-TEXs) could more efficiently induce DC activation and an antigen-specific T-cell immune response than unprocessed TEXs (25) (Figure 2a). This is attributed to the increased level of chemokines, including CCL2, CCL3, CCL4, CCL5, and CCL20 in HS-TEXs, which contribute to the recruitment and stimulation of CD11c^+^ DC and CD4^+^/CD8^+^ T-cells both *in vitro* and *in vivo* (25). Actually, heat stress promotes these chemokines to assemble into lipid rafts, which are then enriched in EVs (25). Nonetheless, the exact mechanism of enhanced immunogenicity of the EVs from stress-treated tumor cells remains unclear.

Wenhai and colleagues (26) found that exosomes released by Rab27a-overexpressed A549 cells had more typical exosomal proteins, including Hsp70 and Hsp90. A mouse model study showed that these EVs induced more BMDCs into mature DCs and then promoted CD4^+^ T-cell proliferation and, thus, gained a stronger antitumor effect (26) (Figure 2b). The authors postulated that the mechanism might be associated with more immunogenic molecules, such as Hsp70 and Hsp90, on modified exosomes (26). Because the postulation is not consistent with the prior (21) and following (27) studies, further research is needed to determine this issue.

Furthermore, TEXs directly decorated with costimulatory molecules also show enhanced immunogenicity. The CD40 ligand (CD40L) can ligate with CD40 expressed on the unique antigen-presenting cells (APCs), including DCs, resulting in enhanced maturation of DCs as well as stronger antitumor CD4 + /CD8 + T-cell immunity (83, 84) (Figure 2c). But, under normal circumstances, CD40L is predominantly expressed on primed T-cells rather than naive T-cells, which may restrict the activation of DCs (85). Researchers successfully designed CD40L-carrying exosomes that possess a stronger ability to trigger maturation of DCs by integrating the features of tumor antigens in TEX, CD40/CD40L targeting DCs, and CD40 signaling (49).

#### Artifactitious EVs From DCs

Dendritic cells are some of the most important antigen-presenting cells. EVs from tumor antigen–stimulated DCs contain pMHC complex, costimulatory molecules, and adhesion molecules and have been proven to be able to elicit MHC-restricted T-cell immunity (50, 52, 86) (Figure 2d). In fact, studies show that the *in vitro* effect of activating T-cells by EVs from APCs is conspicuously weaker as compared to that by corresponding APCs, and the presence of unactivated APCs could apparently enhance EVs’ ability to stimulate T-cells. In other words, *in vivo* activation of T-cells by EVs from APCs entails the assistance of APCs (50, 86, 87). Further studies have revealed that these MHC antigen–bearing exosomes from DCs (DEXs) could be transferred to other DCs, resulting in activation of antigen-specific naïve CD4^+^T-cells (53, 88). Even MHC class II-deficient DCs with costimulatory molecules CD80 and CD86 can adopt DEXs and activate CD4^+^T-cells. In this way, activated DCs may enhance their ability to stimulate T-cells by generating large amounts of DEXs with pMHC complexes (88). The mechanisms of the antigen-presenting roles of EVs have been well reviewed previously (7, 11).

Therefore, we are led to assume an effective adaptive immune activation process in which foreign TEVs can be recognized, processed, and presented by DCs to T-cells. Meanwhile, stimulated DCs release EVs, which causes other unstimulated DCs to engage in antigen presentation and finally enhance their immunostimulatory effects.

## Associated Therapeutic Applications of EVs in Lung Cancer

**FIGURE 3:**
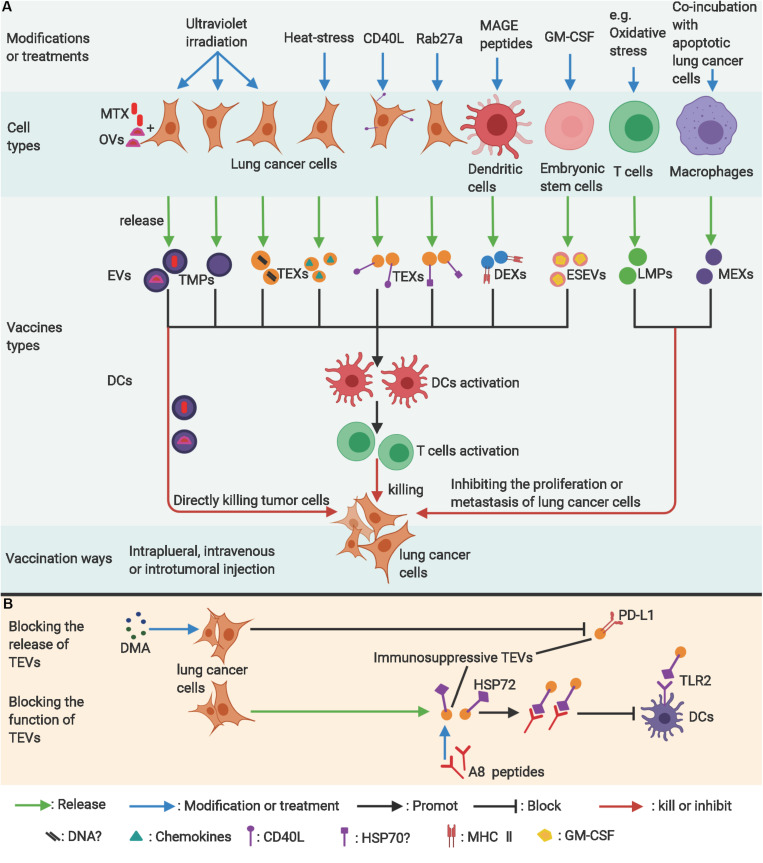
The applications of EVs in lung cancer therapy. **(A)** Modified lung cancer cells, dendritic cells, or embryonic stem cell–derived extracellular vesicles can be used to stimulate antitumor immunity as vaccines. Modified T-cells or macrophage-derived extracellular vesicles can potentially inhibit proliferation of lung cancer cells. **(B)** Blocking the release of exosomes from lung cancer cells (TEXs) or neutralizing the immunosuppressive molecules on TEXs are potentially effective antitumor ways. *?, uncertain; MAGE, melanoma antigen gene; GM-CSF, granulocyte-macrophage colony-stimulating factor; MTX, methotrexate; OVs, Oncolytic virus; TMPs, lung tumor-derived microparticles; TEVs, EVs from lung tumor cells; TEXs, Lung cancer-derived exosomes; DEXs, exosomes from dendritic cells; ESEVs, EVs from embryonic stem cells; LMPs, T-lymphocyte-derived microparticles; MEXs, exosomes derived from macrophages; DMA, Dimethyl amiloride; HSP72, Heat Shock Protein 72; TLR2, toll-like receptor 2*. Figure created with BioRender.com.

Because the mechanisms by which the EVs from tumor or immune cells regulate immunity in lung cancer have been partially, if not fully, understood, we may work out protocols that specifically target relevant mechanisms to minimize EVs’ immunosuppressive effects or maximize their antitumor immunity by modifying EVs or using EVs as immunogenic drug carriers (Figure 3). In the following sections, we discuss existing or ongoing research exploring EV-related applications in the treatment of lung cancer.

### Blocking the Function of EVs

#### Directly Blocking the Production of EVs

Because EVs play significant roles in suppressing anticancer immunity and tumor progression through various pathways (21), blocking the generation or release of EVs is believed to be a feasible way to eliminate their immunosuppressive function. As described above, Hsp72 of TEXs from various tumor cells, including lung cancer, could restrain tumor immune surveillance by activating the MDSCs’ immunosuppressive activity by triggering the TLR2 signaling pathway (21). Dimethyl amiloride (DMA), an inhibitor of the H^+^/Na^+^ and Na^+^/Ca2^+^ channels, and omeprazole, a K^+^/H^+^ ATPase inhibitor, can inhibit the secretion of exosomes (89, 90) (Figure 3B). Therefore, authors have tried to learn if the two drugs can decrease exosome secretion and reverse the activation of MDSCs (21). Chalmin F et al. proved that DMA alone exerted little or no effect although combined therapy (cyclophosphamide plus DMA) could apparently enhance the tumor-inhibitory ability of cyclophosphamide (stimulating T-cell immunity by eliminating regulatory T-cells) by blocking the immunosuppressive function of MDSCs in three non-lung cancer models (21). DMA in combination with CpG could also achieve a synergic effect. Moreover, a human study that used its analog, amiloride (currently used for the treatment of edema or high blood pressure and shown to be able to decrease secretion of exosomes), in 11 patients with colorectal metastatic cancer and high blood pressure showed that MDSCs in the blood of patients had lower phosphorylation of STAT3 and suppressive function (21). Overall, this research proves that blocking the secretion of TEXs could restore certain immune function and enhance the effectiveness of other treatments. Moreover, multiple chemicals that block generation or secretion of EVs were also tried as therapeutic agents for lung cancer (to reverse tolerance to chemotherapies) or other cancers (91). Their potential roles in restoring the immune function of patients with lung cancer need to be further confirmed.

#### Blocking the Interaction Between EVs and Targeting Cells

Apart from decreasing the production of EVs, blocking the contact of EVs with recipient cells might produce similar or better effects. Using an A8 peptide that competitively binds to the domain of membrane HSP70 on tumor-derived exosomes can block the combination of HSP70 with TLR2 and restore the anticancer immune response (27) (Figure 3B). Elevated levels of PD-L1 on DCs induced by exosomes derived from the LLC may decrease the proliferation of CD4^+^T-cells and their differentiation into CD4^+^IFN-γ^+^ Th1 cells but increase the differentiation of Treg cells (19). Anti-PD-L1 antibodies have been shown to significantly reverse this immunosuppressive effect (19). It is also worth noting that the 4T1 exosomes (from breast cancer cells) had a weaker suppressive effect on the CD4^+^T-cells than LLC exosomes. In line with that, treatment with an anti-PD-L1 antibody exerted little restoring effect on the differentiation of CD4^+^T-cells into CD4^+^IFN-γ^+^ Th1 cells (19). These findings suggest that exosomes from different cancer cell types work differently in suppressing immunity. Identification of new molecules/ligands mediating immunosuppression on TEVs will allow us to find novel and more effective therapeutic targets.

### Engineering TEVs Into Immunity-Inducing Vaccines

Many studies find that UV exposure (6, 24), heat treatment (25), or other stresses strengthens the immunogenicity of EVs from tumor cells and, meanwhile, reduces their tumor-promoting properties (Figure 3A). Moreover, gene-modified ligands on EVs can strengthen their ability to target tumor and immune cells and finally enhance tumor-specific immunity (26, 49) (Figure 3A).

#### UV Irradiation

Previous studies proved that UV-exposed tumor-derived microparticles (UV-TMPs) (from melanoma, hepatocellular, colon, and lung carcinoma), rather than naturally secreted TMPs, had the ability to stimulate DC maturation and induce T-cell–dependent antitumor immunity (6, 24). The mechanism underlying the maturation of DCs might involve innate DNA in TMPs implicated in the cGAS/STING pathway in DCs. The activation of the pathway could induce the production of type I IFN (24). With regard to antitumor effects, this UV-TMP vaccine has been shown to be effective only as a prophylactic measure and not as a treatment alternative for preexisting tumors (6, 24). Of note, UV-TMPs were more immunogenic than UV-exposed tumor-derived exosomes (UV-TEXs) and tumor-cell lysates, suggesting that different types of EVs may possess various immunogenicity (24). Understanding this difference and the underlying mechanisms can help us select EVs that work best as the most effective cell-free vaccines against tumors. However, other studies show that UV-processed TMPs are not invariably immunostimulatory (47, 78) as discussed in previous sections.

#### Heat Treatment

In addition, other researchers find that heat-stressed 3LL lung tumor cell–derived exosomes (HS-TEXs) induce more efficient DC activation and antigen-specific T-cell immune response than their unprocessed counterparts (25). This might be ascribed to increased content of various inflammatory chemokine ligands in HS-TEXs, which attract and activate CD11c^+^ DCs and CD4^+^/CD8^+^ T-cells both *in vitro* and *in vivo* (25). Consequently, intratumoral injection of HS-TEXs could more effectively activate specific antitumor immune response than untreated tumor-derived exosomes, thus inhibiting tumor growth and significantly prolonging survival of tumor-bearing mice (25).

#### Rab27a Overexpression

Rab27a is generally seen as a key regulator for exosome secretion from donor cells (92, 93). Johnson and colleagues proved that Rab27a regulates the azurophilic granule exocytosis of neutrophils, which was intimately linked to its microbicidal function (94). Rab27a deficiencies in mice impaired the secretion of myeloperoxidase stimulated by lipopolysaccharides (LPSs) *in vivo* (95). Wenhai et al. (26) find that exosomes from Rab27a-overexpressed A549 cells induce more BMDCs into mature DCs than normal exosomes and, subsequently, promote CD4^+^ T-cell proliferation and exhibit a strong antitumor effect in a mouse model. This mechanism of immune activation might be explained by an increased amount of immunogenic molecules (such as Hsp70 and Hsp90) on the modified exosomes, but this hypothesis needs further verification (26). Intriguingly, other research shows that Rab27a deficiencies decrease the secretion of exosomes and inhibit primary tumor growth and pulmonary dissemination of a metastatic carcinoma (4T1) (93). Actually, exosome secretion does not depend exclusively on Rab27a or Rab27b (12) and may vary with different cells (93). Moreover, Rab27a also participates in the secretion of some non-exosome-associated proteins, which include the metastasis-promoting matrix metalloproteinase 9 (MMP9) (93). The contradictions among these studies involving Rab27 proteins in tumors will not be resolved after fully understanding the role of the Rab27 family.

#### CD40L Modification

It is feasible to directly engineer costimulatory molecules on TEXs to enhance their immunogenicity (49). Researchers attempted to use CD40L-carrying exosomes as a stronger signal to trigger maturation of DCs by combining the features of tumor antigens in TEX and CD40L on exosomes targeting CD40 on DCs (49). As expected, results show that exosomes from CD40L gene-modified 3LL lung cancer cells had stronger ability than normal TEXs in activating the maturation of DCs and then inducing tumor-specific T-cell activation and protracting the survival of mice inoculated with 3LL cells (49).

The foregoing studies show that tumor-derived extracellular vesicles (TEVs) have the potential to induce specific anticancer immunity in either their natural states or artificially engineered forms. On the basis of these findings, we are led to conclude that the centerpieces of all these studies are the tumor-associated antigens on TEVs, and manipulations of TEVs only serve to enhance their interaction with immune cells while reducing their “immunosuppressive components.” However, in fact, antitumor effects of engineered TEVs have been found to be limited in animal research. Moreover, the potential risks of immunosuppression and promoting tumor growth and metastasis may also restrict further application of TEVs as vaccines in clinical practice. On the other hand, even though we can’t guarantee that engineered TEVs are adverse reaction–free, we believe that DCs activated by TEVs and DEXs have no undesirable properties (e.g., favoring tumor growth, angiogenesis, or metastasis) and possess similar or even stronger abilities to stimulate adaptive immunity (52–54, 87). In fact, DC vaccines pretreated with TEVs (28) or DEXs (29, 30, 96) for clinical use are being studied actively.

### DC Vaccines Pretreated With Modified TEVs

A clinical trial employed autologous DCs as multivalent vaccine in 16 patients with stage IA to IIIB NSCLC who had previously received treatment (28). This DC vaccine was stimulated by apoptotic bodies secreted from an irradiated allogeneic NSCLC cell line that overexpressed Her2/neu, CEA, WT1, Mage2, and surviving cells (28) (Figure 3A). Results show that the vaccine was well tolerated although no apparent benefits in clinical outcomes were achieved except in two individuals (28). In addition, though specific and non-specific immunologic responses to vaccines could be found in some patients, there was no significant association between immune responses (as measured by IFN-γ ELISPOT) and clinical outcomes. The result might be attributed to the use of an improper indicator for monitoring immune activation (28). Anyway, this research proves that the DC vaccine is feasible and does have certain biological activities. The study provides some useful information for improving the design of future studies. Indeed, use of multivalent antigens from modified allogeneic tumors and the heterogenicity of patients might be two major causes responsible for the limited efficacy (28).

### Engineering DEXs Vaccines

The feasibility of producing autologous DEXs loaded with specific MAGE peptides and the tolerance and safety of the vaccine in MAGE^+^ NSCLC patients has been proven by a phase I clinical trial (29) (Figure 3A). Only grade 1–2 adverse events were monitored, and a few patients with advanced NSCLC achieved a long PFS after immunization (29). Activation of NK cells could be observed in some patients, but no significant increase was found in antigen-specific T-cell activity, which might be ascribed to increased CD4^+^CD25^+^ T-regulatory T-cells (29). Meanwhile, another phase I study using MAGE-loaded DEXs in patients with MAGE3^+^ advanced melanoma yielded similar or more optimistic results (96). Encouraged by these successes, the researchers initiated a phase II clinical trial with second-generation DEXs (IFN-γ-DEXs, derived from IFN-γ-stimulated mature dendritic cells) loaded with MHC class I- and class II-restricted MAGE antigens as vaccines (30). This vaccine was designed to enhance both NK and T-cell immune functions and to explore whether it could improve the clinical outcomes of chemotherapy-stabilized/responding NSCLC patients (30). Results suggest that IFN-γ-DEXs could enhance the functions of NKp30-dependent NK cells but failed to significantly induce the activation of antigen-specific T-cells (30). Further studies indicate that the functional enhancement of NK cells was correlated with prolonged PFS of the patients, and this enhancement depended on the reaction of NKp30 on NK cells with its ligand BAG6 on the IFN-γ-DEXs (30). It is noteworthy that previous melanoma studies showed that NK activation, induced by DEX from immature DCs, relied on NKG2DL and the IL-15Ralpha signaling pathway (96, 97). Questions remain to be answered regarding the limited effects of DEX vaccines: whether other antigens could be engineered on DEXs to arouse stronger antitumor immunity and whether combined treatment of DEX vaccines and immune-checkpoint blockers generate stronger immune synergy (30).

### Engineering Embryonic Stem Cell (ESC)–Derived Exosomes as Vaccines

Common antigens between tumor and embryonic cells are the immunological basis for using embryonic cells as antitumor vaccines (98). An interesting attempt was conducted to stimulate antitumor immunity by using exosomes from ESCs, which may express similar carcinoembryonic antigens as some tumor cell types (31) (Figure 3A). Vaccination with exosomes from granulocyte-macrophage colony-stimulating factor (GM-CSF)–expressing murine embryonic stem cells could prevent the growth of implanted LLC lung adenocarcinoma, B16-F10 melanoma, MC-38 colon adenocarcinoma, and 4T1 mammary carcinoma but not E0771 medullary breast adenocarcinoma in allogeneic mice (31). This vaccine resulted in increased tumor cell-specific CD8^+^ T-cells and a decreased percentage of MDSCs in the spleen and raised the ratio of CD8^+^ T-effector cells to Tregs in the tumor (31). This ESEV vaccine could avoid the risk of embryomas/teratomas caused by the whole-ESC vaccine. More studies are needed to ascertain the common antigens between the ESCs and lung cancer cells for the future application of this vaccine.

### Functioning as an Immunogenic Drug–Delivery System for Its Specific Tumor Tropism

Tumor-derived EVs have a specific tumor tissue/cell tropism (99). A *in vivo* study using fluoresce DiIC18 to label the EVs showed that paclitaxel (PTX)-encapsulated EVs (EV-PTX) could transform PTX-induced systemic inflammation to peritumoral inflammation (32). Some reviews (100–102) mention that EVs can be made into an effective drug-delivery system for cancer therapy by modifying their tropism.

Human EVs from lung cancer cells have been shown to serve as vehicles for delivering oncolytic virus (OVs) and PTX to reduce tumor growth in nude mice with compromised immune systems (33) (Figure 3A). Compared to OVs alone or OVs + PTX, EVs encapsulation could apparently increase infectious titer or transduction ratio of OVs in lung cancer cells and showed a stronger tumor-suppressing effect (33). Further studies proved that the murine lung cancer cell–derived EVs containing OVs and PTX, but not EVs alone, could induce immunogenic death of cancer cells *in vitro* as indicated by the increased expression of calreticulin on the cell surface and the extracellular release of ATP (32). Treatment with a virus, EV-Virus and EV-Virus-PTX, could selectively induce peritumoral inflammation, although not systemic inflammation, as indicated by increased infiltration of TILs (32). The EV-virus could induce stronger cytotoxic immunity than the virus alone, which might be because EV encapsulation may protect the virus from immune surveillance (32). Notably, a systemic inflammatory reaction would take place upon treatment with PTX alone although EV encapsulation could significantly prevent the systemic reaction, suggesting that EVs derived from tumor cells do have strong specific cancer tissue tropism (32).

Recently, in a phase I clinical trial, we intrapleurally administered ATMPs-MTX to advanced lung cancer patients with malignant pleural effusion and produced encouraging results (6). TMPs could be intrapleurally injected into mice quickly, and most of TMPs stayed or assembled in the lungs and tumor tissues. TMPs-MTX show apparent tumor tropism and exert cytotoxicity on tumor cells and tumor-associated macrophages but not on T-cells, and apoptotic tumor cells treated by TMPs-MTX could activate DCs both *in vitro* and *in vivo* (6). Intrapleural infusion of ATMPs-MTX into patients has been shown to be safe, well tolerated, and clinically beneficial without grade 3 or higher toxic effects. In particular, it could create an immune-activated intrapleural microenvironment as indicated by increased effector immune cells and cytokines and decreased suppressive immune cells (6).

Collectively, certain molecules contained in TEVs could make them more stable in blood and help them target tumor issues more accurately, thereby making TEVs an excellent delivery carrier. Enhanced tropism conferred by OVs (32) and modified ligands or use of proper local administration routes (e.g., intrapleural injection) in combination with encapsulated chemotherapeutic agents can effectively enhance the antitumor efficacy of TEVs, partially by activating antitumor immunity.

### Modified EVs From T-Cells and Macrophages Directly Inhibit Lung Cancer

Intriguingly, microparticles derived from three types of T-lymphocytes (LMPs), including hominal peripheral T-lymphocytes stimulated by various stimuli (apoptosis, cell division, or oxidative stress), could inhibit the proliferation of various tumors, including lung cancer *in vitro* and *in vivo* (35) (Figure 3A). The mechanism of these non-species-specific antitumor effects is associated with arrest of the cell cycle at G_0_/G_1_ for upregulated expression of cyclin-dependent kinase inhibitors (CDKIs) in lung cancer cells, consistent with which these microparticles were ineffective for quiescent cancer cells (35). Additionally, researchers have not reached a consensus on the effect of the LMPs on angiogenesis under different stimuli (34, 103, 104). Moreover, researchers also found that exosomes from activated T-cells could enhance the invasiveness of 3LL cancer cells by upregulating the expression of MMP9 through the Fas/FasL signaling pathway (105). In summary, better understanding of how EVs from T-cells under various conditions work differently on lung cancer cells in the future is a prerequisite for developing them as an effective treatment for lung cancer.

A recent study shows that exosomes derived from macrophages (MEXs), stimulated with UV-induced apoptotic lung cancer cells, could inhibit lung metastasis (106) (Figure 3A). The mechanism involves the transportation of PTEN protein from MEXs to cancer cells, thereby inhibiting epithelial-mesenchymal transition (106).

Reviewing these related studies can give us a holistic view of the roles of EVs from immune cells on cancer cells and helps us further study the mechanism by which EVs from vaccine-stimulated T-cells (or other immune cells) kill or inhibit tumors.

## Conclusion and Future Directions

In summary, EVs, as a communication medium between cancer and immune cells, plays significant roles in lung cancer immunity. Modification of tumor or immune cells can alter the immunoregulatory function of EVs even from immunosuppressive roles to immunostimulatory ones. Treatment of lung cancer can be approached by targeting the mechanisms by which EVs mediate lung cancer immunity.

Further exploration of immunoregulatory mechanisms and therapeutic applications of EVs in lung cancer is essential, and existing studies have shown good prospects. On the basis of the aforementioned literature focusing on EVs’ roles in immunoregulation and therapy of lung cancer, the authors believe future research efforts should be directed to the following fields.

First, so far, there is still no definitive markers and effective isolation methods for different EV subtypes (9, 107). This means that certain subtypes of EVs in existing studies are most likely to be a mixture of different EV subsets in various proportions. Identifying them was and will be the greatest challenge for the study of EVs. Second, most of the studies focus on the immunoregulatory roles of exosomes and microparticles rather than apoptotic bodies. Generally speaking, they all play an immunosuppressive role during the development of lung cancer, and all of them can be modified in various ways to induce antitumor immunity. Nonetheless, few studies compared the immunoregulatory ability of these EV subsets in cancer. For instance, H22 hepatocarcinoma cell–derived microparticles (H22-MPs) are shown to be more immunogenic than tumor cell lysates and tumor cell–derived exosomes in inducing T-cell-dependent antitumor immunity (41). Of note, though the microparticles, exosomes, and tumor cell lysates in that study were collected from the same number of H22 cells, the pretreatment of donor H22 cells was different, which might impact the immunogenicity of these EV subsets. Therefore, more efforts are needed to compare the immunogenicity of these EV subsets to figure out which type of EV subsets works best as an antitumor vaccine. Third, previous research focuses principally on the phenotypic changes of recipient cells of EVs and not on the underlying mechanisms (19, 26, 78). Further studies should identify potential substances on EVs and associated signaling pathways underlying those functions. Fourth, the accurate assessment of potential risks associated with the application of TEX vaccines, such as metastasis (16) and/or angiogenesis (17), will also determine whether they will be safely put into clinical use. Fifth, Some researchers proved that UV-TMPs could induce differentiation of suppressive M2 macrophages and inhibit antitumor immunity (47, 78), and others proved that UV-TMPs could activate DCs and stimulate antitumor immunity (6, 24). Further studies should look into the functional changes of both DCs and macrophages in the same subject to ascertain the final immunoregulatory outcomes of UV-TMPs. Sixth, effective immune activation entails three prerequisites, including antigen presentation, activation of CD4 + /CD8 + T-cells, and persistent stimulation of cytokines. The immunoregulatory network is so complicated that EV vaccines used for human lung cancer therapy are far from satisfactory (29, 30). ATMPs in combination with MTX have shown encouraging clinical benefits in advanced lung cancer patients with malignant pleural effusion (6). Therefore, EVs in combination with other therapies, such as PD-1/PD-L1 blockade or radiotherapy and so on, should be tried to explore optimal therapeutic regimens for lung cancer.

## Author Contributions

ZY, JF, and YJ participated in the study design and the manuscript preparation. ZY and JF wrote the manuscript. JX, YL, and MZ contributed to figure preparation. LD and TL made the tables. SW, WG, and YJ were involved in manuscript revision. All authors read and approved the final manuscript.

## Conflict of Interest

The authors declare that the research was conducted in the absence of any commercial or financial relationships that could be construed as a potential conflict of interest.

## Abbreviations

AICD: activation-induced cell death
APCs: antigen-presenting cells
APO2L: Apoptosis ligand 2
ATMP-MTX: ATMPs loaded with methotrexate
ATMPs: autologous tumor-derived microparticles
BAG6: BCL2-associated athanogene 6
CAR: chimeric antigen receptor
CCL: chemokine (C-C motif) ligand
CCR: C-C motif chemokine receptor
CD40L: CD40 ligand
CDKIs: cyclin-dependent kinase inhibitors
CEA: carcinoembryonic antigen
cGAS: Cyclic GMP-AMP synthase
CTLA4: cytotoxic lymphocyte antigen 4
DCs: dendritic cells
DEXs: exosomes from dendritic cells
DMA: Dimethyl amiloride
ESCs: embryonic stem cells
ESEVs: EVs from embryonic stem cells
ESEXs: exosomes from embryonic stem cells
EV-PTX: PTX encapsulated EVs
EVs: extracellular vesicles
FasL: Fas ligand
GM-CSF: granulocyte-macrophage colony-stimulating factor
Her2/neu: human epidermal growth factor receptor 2
HSP70/72: Heat Shock Protein 70/72
HS-TEXs: Heat-stressed lung tumor cell-derived exosomes
IDO: indoleamine 2,3-dioxygenase
IL-1β: interleukin-1β
IL-6: interleukin-6
iNOS: inducible nitric oxide synthase
ISEV: The International Society for Extracellular Vesicles
LEVs: EVs from T lymphocytes
LLC: Lewis lung cancer
LMPs: T lymphocytes-derived microparticles
MAGE: melanoma antigen gene
MDSCs: myeloid-derived suppressor cells
MEXs: exosomes derived from macrophages
MHC I/II: major histocompatibility complex I/II
MMP9: matrix metalloproteinase 9
MTX: methotrexate
MVBs: multivesicular bodies
MyD88: myeloid differentiation factor 88
NKp30: natural killer (NK) cell receptor
NSCLC: non-small cell lung cancer
OVs: Oncolytic virus
PBMCs: peripheral blood mononuclear cells
PD-1: programmed death-1
PD-L1: programmed death-ligand 1
pMHC II: peptide-MHC II
SOCS3: suppressors of cytokine signaling 3
STAT3: signal transducer and activator of transcription 3
STING: stimulator of interferon gene
TABs: lung tumor-derived apoptotic bodies
TEVs: EVs from lung tumor cells
TEXs: Lung cancer-derived exosomes
TGF-β: transforming growth factor-β
TIDC: tumor-infiltrating DCs
TIME: tumor immune-microenvironment
TLR: toll-like receptor
TNF-α: tumor necrosis factor-α
TRAIL: tumor necrosis factor-related apoptosis-inducing ligand
Treg cells: regulatory T-cells
UV: ultraviolet
UV-TEXs: UV-exposed tumor-derived exosomes
UV-TMPs: UV-exposed tumor-derived microparticles
VEGF: vascular endothelial growth factor
WT1: Wilms’, tumor gene 1.
